# Patient‐Reported Feedback Suggests an Alternative Sweet Spot for Deep Brain Stimulation Programming in Essential Tremor

**DOI:** 10.1002/mds.70228

**Published:** 2026-03-06

**Authors:** Sophia Peschke, Jing Dong, Angelina Kirschner, Johannes Off, Juhi Shaik, Carla Palleis, Jan Hinnerk Mehrkens, Elisabeth Kaufmann, Maximilian Scherer, Thomas Koeglsperger

**Affiliations:** ^1^ Department of Neurology LMU University Hospital, LMU Munich Munich Germany; ^2^ Department of Neurosurgery LMU University Hospital, LMU Munich Munich Germany; ^3^ Department of Translational Brain Research German Center for Neurodegenerative Diseases (DZNE) Munich Germany

**Keywords:** connectomics, deep brain stimulation (DBS), essential tremor, Visual Analogue Scale (VAS)

## Abstract

**Background:**

Deep brain stimulation (DBS) of the ventral intermediate nucleus (VIM) and caudal zona incerta is an established therapy for essential tremor (ET). Clinical outcomes depend on precise electrode placement and optimal stimulation parameters. Effective programming must balance tremor suppression with side‐effect risk, yet systematic incorporation of patient‐reported feedback remains limited.

**Objective:**

The aim was to assess whether subjective patient feedback, quantified via a Visual Analogue Scale (VAS), can guide DBS programming for effective tremor control.

**Methods:**

In 15 VIM‐DBS patients, 1253 unique stimulation settings were collected, each rated using a VAS reflecting perceived clinical benefit. Associated volumes of tissue activated were mapped and analyzed. VAS‐optimized settings were compared to standard‐of‐care (SoC) programming. Voxel‐wise permutation statistics identified stimulation sweet and sour spots, whereas structural and functional connectivity analyses determined neural correlates of subjective benefit.

**Results:**

VAS‐optimized stimulation achieved tremor suppression comparable to SoC settings but with lower energy consumption. Sweet spots correlated with high VAS ratings localized to the dorsal VIM, whereas sour spots were ventral. Connectivity between sweet spots and prefrontal, frontal, and insular regions positively correlated with perceived benefit.

**Conclusions:**

Integrating patient‐reported feedback offers a structured, individualized approach to DBS optimization in ET. VAS‐guided programming identifies patient‐specific sweet spots and delineates connectivity profiles associated with clinical benefit. Particularly, VAS‐derived sweet spots were more dorsal than previously suggested targets, highlighting the importance of incorporating subjective feedback to refine optimal stimulation regions. © 2026 The Author(s). *Movement Disorders* published by Wiley Periodicals LLC on behalf of International Parkinson and Movement Disorder Society.

## Graphical abstract

(1) To sample subjective patient rating using the Visual Analogue Scale (VAS), random stimulation parameters were presented to the study subjects. (2) Next, corresponding volumes of tissue activated (VTA) were generated and paired with VAS values. (3) These paired VAS/VTA data were aggregated voxel‐wise to construct a heat map representing the “subjective sweet and sour spot.” (4) Finally, connectomes were seeded from the identified sweet spots.

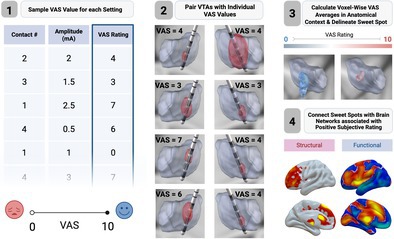

Essential tremor (ET) is a common movement disorder, characterized by progressive, bilateral action and postural tremor predominantly affecting the upper limbs but also involving the head, voice, trunk, and, less frequently, the lower limbs.^1^ For patients with medically refractory symptoms, deep brain stimulation (DBS) has emerged as a safe and effective long‐term treatment option, achieving tremor reductions of 66% to 78% at 1 year after bilateral implantation, with some individuals experiencing up to 90% improvement.^2^, ^3^, ^4^

Optimal DBS outcomes depend on proper patient selection, accurate electrode placement, and systematic postoperative programming.^5^, ^6^ Directional DBS electrodes allow finer control of current spread, improving efficacy and side‐effect profiles.^7^ However, this advance increases programming complexity due to more possible parameter combinations.^8^ Thus, efficient strategies are needed to optimize stimulation parameters and lead configurations.

Selecting the most effective and well‐tolerated contact remains central to DBS programming. The “sweet spot” for tremor suppression is still being refined. The ventral intermediate nucleus (VIM) of the thalamus—connecting the primary motor cortex and the contralateral dentate nucleus via the dentato‐rubro‐thalamic tract (DRTT)—has been the main ET‐DBS target.^9^, ^10^, ^11^ However, similar benefits have been achieved by stimulating adjacent regions like the posterior subthalamic area (PSA) and caudal zona incerta (cZi). A few studies have directly compared efficacy and side‐effect profiles across DBS targets. Evidence indicates that proximity to the DRTT predicts outcomes better than anatomical location, with closer stimulation linked to lower tremor‐suppression thresholds.^12^, ^13^ This supports the idea that effective sites modulate the shared DRTT pathway spanning the red nucleus, zona incerta, PSA, and VIM.^14^, ^15^, ^16^, ^17^, ^18^

Although some studies include patient‐reported outcomes via activities of daily living (ADL) assessments,^11^, ^15^, ^19^, ^20^ a few examine patients' subjective perceptions, which are crucial to treatment success. Despite technological advances, programming routines that integrate patient feedback remain scarce.^8^, ^21^ Previously, we showed that a Visual Analogue Scale (VAS)–based programming approach in Parkinson's disease achieved outcomes comparable to standard methods while identifying stimulation networks linked to patient‐rated benefit.^22^, ^23^ In the present study, we extend this VAS‐based approach to VIM‐DBS for ET, aiming to investigate its feasibility, its effectiveness in guiding stimulation programming, and its potential to elucidate patient‐specific stimulation sweet spots and associated connectivity profiles.

## Patients and Methods

### Study Participants

All study procedures were approved by the local ethics committee of Ludwig Maximilian University of Munich, Germany (approval no. 18‐809), and written informed consent was obtained from all participants prior to inclusion according to the Declaration of Helsinki. Patients with ET who had undergone DBS targeting the VIM were recruited between June 2022 and December 2023 during routine visits to our movement disorders outpatient clinic. Participants were eligible if they met the following criteria: (1) a diagnosis of ET according to the German Neurological Society (DGN) guidelines^24^ and (2) treatment with VIM‐DBS. Additional inclusion criteria required that patients must have been implanted for at least 1 year and that their DBS settings have remained stable for a minimum of 3 months prior to study participation. Patients with manifest dementia or uncontrolled psychiatric disorders were excluded. All patients underwent awake, staged bilateral implantation targeting the VIM using standard stereotactic coordinates with magnetic resonance imaging (MRI)‐based anatomical adjustments, and the final target was refined intraoperatively through systematic macrostimulation to avoid side effects and ensure adequate tremor control.

### Study Visit and VAS Rating

The study protocol has been described previously,^23^ with details in the Supplementary Material. Tremor severity was assessed using the Fahn‐Tolosa‐Marin Tremor Rating Scale (FTMTRS) before and after a 60‐s bilateral DBS washout. VAS‐based reprogramming was then performed, testing each hemisphere separately with the contralateral side off. Individual contacts were stimulated at 0 to 3.0 mA randomly (Table S1; Fig. S4), and blinded patients rated overall DBS effects on a scale of 0 to 10 (0 = worst effect, 10 = best effect) within 10 to 15 s.^22^, ^23^, ^25^ Contacts causing intolerable side effects were excluded, with a 10‐s washout between settings. The best‐rated setting per hemisphere (Pulse Width (PW) 60 μs, 130 Hz) was selected, followed by final FTMTRS assessment and restoration of original settings.

### Localization of DBS Leads

Preoperative imaging (T1‐ and T2‐weighted MRI) and postoperative computed tomography (CT) scans were processed for electrode localization using the Lead‐DBS toolbox (www.lead-dbs.org).^26^ Postoperative CT images were linearly co‐registered to the corresponding preoperative MRI using Advanced Normalization Tools.^27^ Native‐space images were subsequently normalized to Montreal Neurological Institute (MNI) space via a three‐step affine registration pipeline, with additional manual refinement of the atlas fit performed where necessary^28^. Correction for brain shift and pneumocephalus resulting from surgery was applied using the Schönecker brain shift correction algorithm implemented in Lead‐DBS.^29^ All registration steps were carefully inspected and manually adjusted to ensure accurate alignment. Electrode trajectories were initially reconstructed using the PaCER algorithm,^30^ with manual refinement performed as needed to optimize anatomical fidelity. In patients implanted with directional leads, rotational orientation was assessed and corrected using the DiODe algorithm.^31^ Anatomical segmentations were defined using the DISTAL atlas.^32^

### Volume of Tissue Activated Estimation

The volume of tissue activated (VTA) was estimated using a finite element method–based model implemented in the Lead‐DBS toolbox. To accurately model the distribution of the electric field, subcortical gray matter nuclei were defined using the DISTAL atlas. Consistent with established protocols, tissue conductivities were set to 0.33 S/m for gray matter and 0.14 S/m for white matter.^33^ The electric fields (E‐fields) were calculated using the SimBio/FieldTrip computational pipeline within Lead‐DBS. A threshold of 0.2 V/mm was applied to delineate the VTA boundaries, following similar methodologies used in previous studies.^33^ For each stimulation configuration, the corresponding VTA was generated and then associated with the corresponding VAS rating.

### Sweet‐Spot Analysis

Sweet‐spot mapping was performed using Sweetspot Explorer^34^ to identify neuroanatomical regions within the VIM associated with subjective patient ratings. First, VAS scores for the entire cohort were normalized within each patient using *z* scores; the resulting standardized values were then incorporated into the analysis. VTAs were generated for each stimulation setting (ie, combinations of contact and amplitude) and thresholded at an electric field magnitude >0.2 V/mm. To ensure meaningful inclusion of stimulation‐affected regions, only voxels that were covered by at least 20% of the VTAs were retained for subsequent analyses, minimizing the inclusion of areas with negligible or no stimulation coverage.^26^, ^33^ A nonlinear transformation was applied to mirror the left hemisphere VTAs onto the right hemisphere. For each voxel (0.22 × 0.22 × 0.22 mm), all stimulation settings whose VTA encompassed that voxel contributed their corresponding VAS scores. These scores were then subjected to a voxel‐wise one‐sample *t* test against zero, yielding a statistical t‐map representing regions where stimulation effects significantly differed from zero. Amplitudes were corrected to account for larger VTAs generated during high‐amplitude stimulation effect more on stimulation maps as they provide more voxels as described previously.^26^, ^33^ The *T*‐values were color coded, and the significance was determined at an *α*‐level of 0.05 as described.^35^ We assessed the voxel‐wise sweet‐spot model's effectiveness and robustness using a leave‐one‐cohort‐out cross‐validation in which each patient's stimulation settings formed a cohort, the significant t‐map served as the overlap metric, the model was recalculated with one cohort removed in each iteration, and the left‐out cohort's predictive value was computed as the mean *t*‐value of voxels where its VTA(s) intersected the significant t‐map. VTA overlaps were generated for each hemisphere and condition (VAS‐ and standard‐of‐care [SoC]‐based stimulation) and transformed to a common MNI space. For each condition, VTAs were binarized and summed voxel‐wise to construct overlap maps (n‐maps), where each voxel value represented the number of VTAs covering that voxel. To visualize both the overall stimulation extent and a potential convergence zone, we defined (1) a union mask including voxels covered by at least one VTA (n ≥ 1) and (2) a core overlap mask including voxels covered by at least 50% of VTAs (n > 50%).

### Connectivity Estimation

To delineate the network correlates of subjective benefit, we performed whole‐brain normative structural and functional connectivity analyses using each individual VTA as a seed region. Functional connectivity was assessed using the resting‐state functional group connectome of 1000 genomics superstruct project (GSP) healthy subjects,^36^ whereas structural connectivity was estimated using a structural group connectome of 985 subjects from the Human Connectome Project.^37^ For each VTA, an individual whole‐brain connectivity profile was computed, representing either the probability of structural connections or the strength of functional coupling to all other voxels in the brain. Three types of group‐level connectivity maps were then generated in accordance with the Lead‐DBS Network Mapping Explorer framework. First, weighted average maps (A‐maps) were calculated by weighting each patient's connectivity profile by the corresponding VAS score, thereby highlighting regions preferentially connected to VTAs associated with greater subjective improvement. Second, voxel‐wise correlations between connectivity strength and VAS ratings were computed across patients using Spearman's rank correlation, yielding correlation maps (R‐maps) with associated significance testing (permutation‐based correction for multiple comparisons). Finally, a combined map (C‐map) was derived by retaining only voxels consistently identified in both the A‐map and the R‐map, with the sign of the correlation (positive or negative) preserved. These maps delineate the structural and functional networks whose connectivity with the stimulation site was most reliably associated with higher or lower subjective ratings of stimulation.

## Results

### Characterization of the Study's Participants

In total, data from 15 study participants was analyzed (Table 1; Table S2), as 1 of 16 enrolled patients had to be excluded at a later time point due to severe symptoms when the stimulation was turned OFF. Five were women, with a mean age of 71.0 ± 8.1 years. The average disease duration for the entire group was 30.8 ± 16.9 years, and the average duration of DBS treatment was 7.4 ± 5.4 years. The FTMTRS (part A + B) scores prior to the study visit were 44.5 ± 4.7 (mean ± standard error of the mean [SEM]) in the OFF setting. Four patients had unsegmented electrodes, and 11 had directional electrodes. These patients were exposed to varying amplitudes ranging from 0.5 to 3.0 mA in 0.5‐mA increments at each ring level (additionally the stimulation was switched off in between for each side once), while each contact was tested separately for patients with segmented electrodes. This resulted in 48 different combinations per side for each patient with 8‐contact leads and 24 combinations per side for those with 4‐contact leads. Each set of combinations is referred to as a cohort. The dataset included 15 patients in total: 4 with 4‐contact electrodes and 11 with 8‐contact electrodes. One patient had a single electrode implanted, whereas all others had bilateral (left and right) electrode placements. This configuration resulted in 204 individual contacts tested. Across these contacts, 1253 unique stimulation settings were applied. The average VAS score recorded for the dataset was 4.46 ± 2.83 (mean ± SEM).

**TABLE 1:** Participant demographics and DBS characteristics

Characteristic	Value
Total participants analyzed, n	15
Sex, female, n (%)	5 (33.3%)
Age (yr) (mean ± SD)	71.0 ± 8.1
Disease duration (yr) (mean ± SD)	30.8 ± 16.9
Duration of DBS therapy (yr) (mean ± SD)	7.4 ± 5.4
Electrode type	
4‐Contact (unsegmented), n	4
8‐Contact (directional), n	11
Electrode laterality	
Unilateral implantation, n	1
Bilateral implantation, n	14

*Notes*: The table summarizes total participants, sex distribution, age, disease duration, and duration of DBS therapy (mean ± SD). Electrode type (4‐contact unsegmented or 8‐contact directional) and laterality of implantation (unilateral or bilateral) are also provided.

Abbreviations: DBS, deep brain stimulation; SD, standard deviation.

### VAS‐Based Programming Results in Similar Acute Effects

**FIG. 1:**
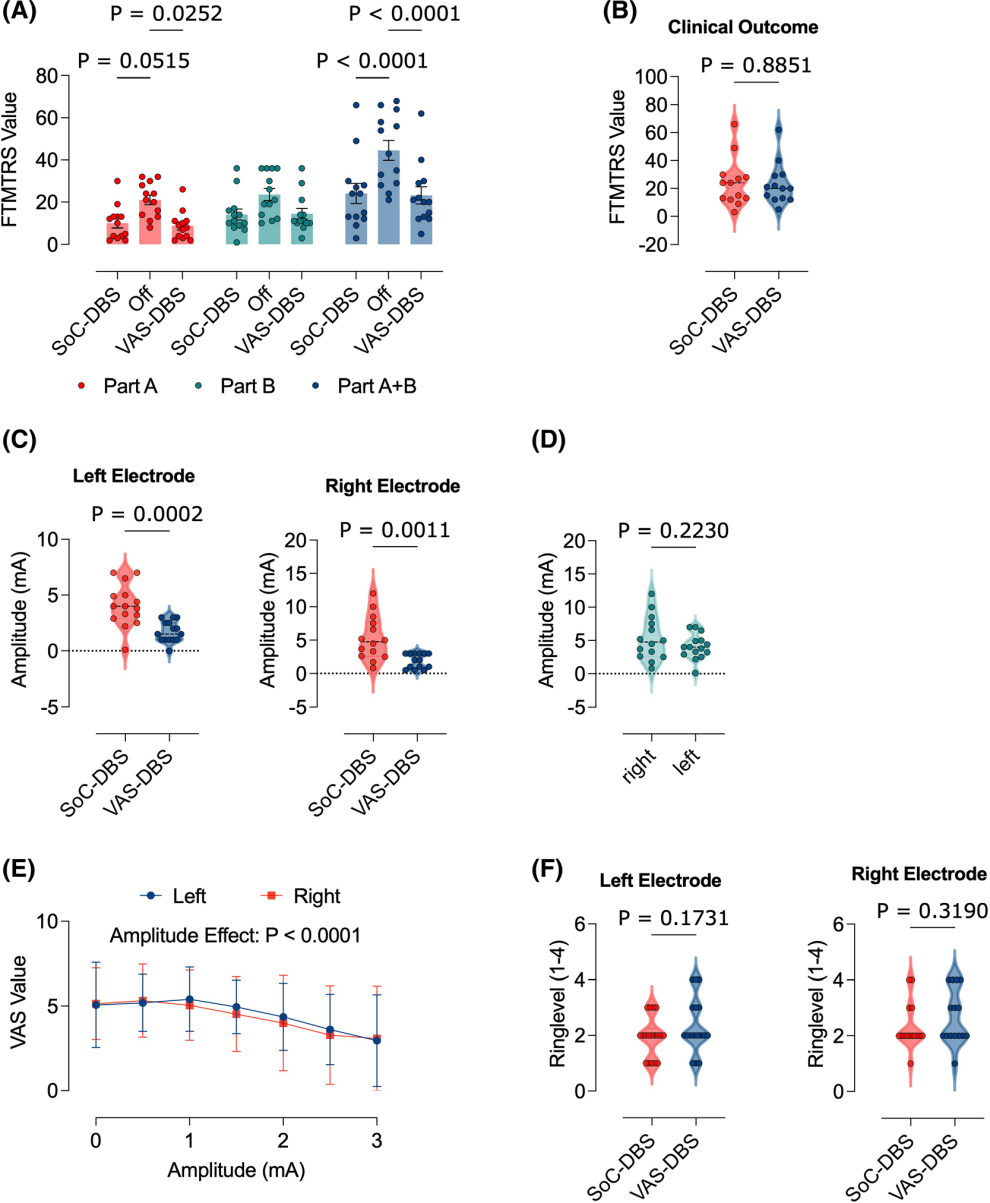
Visual Analogue Scale (VAS)–based programming results in effective short‐term tremor control. (**A**) Bar graph illustrating FTMTRS values (parts A, B, and A + B) under the chronic deep brain stimulation (DBS) settings before VAS assessment (standard of care [SoC]), in response to switching off DBS (OFF) and under VAS‐based DBS setting (VAS‐DBS). (**B**) Graphs illustrating the before and after FTMTRS values under SoC conditions and VAS‐DBS for each individual study participant. (**C**) Graphs illustrating the amplitude (mA) under SoC conditions and VAS‐DBS for each individual study participant (left and right graphs). (**D**) The VAS‐derived amplitudes were indifferent between the right and left leads (right graph). (**E**) Graph illustrating the average VAS values across all study subjects as a function of the stimulation current, demonstrating a peak at 1 mA and a decline with higher amplitudes. (**F**) Graphs illustrating the before and after electrode ring levels under SoC conditions and VAS‐DBS for each individual study participant. Statistical comparisons between OFF, standard‐of‐care DBS (SoC‐DBS), and VAS‐guided DBS (VAS‐DBS) conditions were performed using a two‐way repeated‐measures ANOVA (analysis of variance) with stimulation condition (OFF, SoC‐DBS, VAS‐DBS) and subscale (A, B) as factors. Tukey's post hoc tests were applied to correct for multiple comparisons, with statistical significance defined as *P* < 0.05 in panel A. Pair‐wise comparisons between two independent groups were performed using unpaired *t* tests in panels B, C, and E. VAS scores were analyzed as a function of DBS amplitude using a two‐way repeated‐measures ANOVA with amplitude as the within‐subject factor in panel E. VAS–amplitude correlations were analyzed in panel F using a separate two‐way repeated‐measures ANOVA with amplitude as the within‐subject factor to assess dose–response characteristics. Effect sizes and 95% confidence intervals were calculated for all relevant contrasts. Data is shown as mean ± SEM (standard error of the mean). [Color figure can be viewed at wileyonlinelibrary.com]

FTMTRS scores were compared under standard‐of‐care DBS (SoC‐DBS), stimulator off (OFF), and the best‐rated VAS‐based setting (VAS‐DBS). Tremor reoccurred immediately when the stimulator was switched off, with significant differences in part A and part A + B scores (Fig. 1A,B). Two‐way analysis of variance (ANOVA) showed significant effects of stimulation (*F*(2,108) = 18.00, *P* < 0.0001), with no significant interaction (*F*(4,108) = 1.185, *P* = 0.32). Post hoc tests indicated that both SoC‐DBS and VAS‐DBS improved tremor compared with OFF, whereas SoC‐DBS and VAS‐DBS did not differ (mean difference: 0.92, 95% confidence interval: −10.1 to 11.98, *P* = 0.98), supporting comparable short‐term efficacy. Stimulation amplitudes were lower in VAS‐DBS compared with SoC‐DBS on both hemispheres (unpaired *t* test, *P* < 0.05), with no interhemispheric differences (Fig. 1C,D). Correlating VAS scores across contacts revealed peaks at 1 mA (left) and 0.5 mA (right), with higher amplitudes leading to decreased VAS scores (*F*(6,162) = 9.65, *P* < 0.0001) and no left–right differences (*F*(1, 27) = 0.06, *P* = 0.81) (Fig. 1E). Ring‐level heights (1–4) varied across cases, but no group‐level differences were found between SoC‐DBS and VAS‐DBS (unpaired *t* test, *P* > 0.05) (Fig. 1F).

### Optimal Stimulation Regions Predict Subjective Patient Feedback

**FIG. 2:**
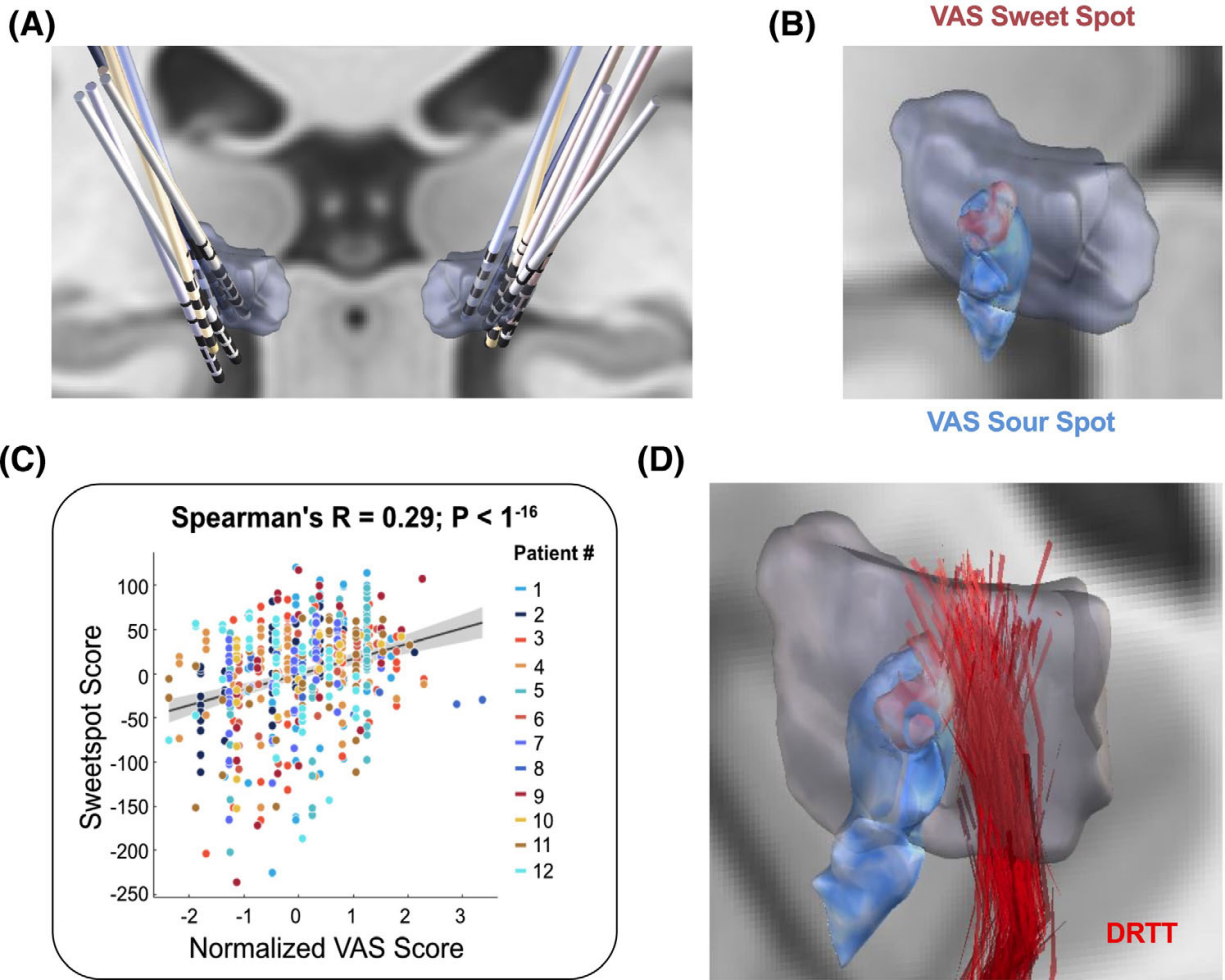
Subjective sweet and sour spots segregate to distinct ventral intermediate nucleus (VIM) subregions. (**A**) Image illustrating the reconstruction of electrodes for each individual study participant. (**B**) Image illustrating the voxel‐wise distribution of positively (red) and negatively (blue) rated voxels across the VIM (*T*‐values, *P* > 0.05). (**C**) Graph demonstrating a positive correlation between normalized Visual Analogue Scale (VAS) scores and the sweet‐spot score. (**D**) Image illustrating the spatial relation of the VAS sweet and sour spots with the dentato‐rubro‐thalamic tract (DRTT). [Color figure can be viewed at wileyonlinelibrary.com]

To identify brain regions associated with subjective sweet and sour spots, DBS electrode reconstructions were analyzed across all participants (Fig. 2A; Fig. S2). VTAs were correlated with individual VAS scores to map regions linked to favorable and unfavorable subjective ratings. Voxels corresponding to higher VAS ratings clustered within the dorsal (cranial) portion of the VIM, although this putative sweet spot did not reach statistical significance (*P* > 0.05) in our leave‐one‐cohort‐out analysis. In contrast, a significant sour spot (*P* < 0.05) was identified in the posteroventral (caudal) VIM and adjacent subthalamic region (Fig. 2B; Fig. S1). The center of mass for the sweet spots in both hemispheres combined was at MNI coordinates (*x*, *y*, *z*) + 15.86, −14.60, 4.04 mm, and the sour spot was at +15.64, −18.12, −2.78 mm. Using a leave‐one‐cohort‐out approach, all cohorts except one were used to train the model in each iteration, and the held‐out cohort was then predicted to test whether VAS scores could improve the prediction of stimulation effectiveness. The results showed a positive correlation between VAS scores and the degree of overlap between the VTA and the sweet‐spot map (*R* = 0.29, *P* < 1^−16^) (Fig. 2C). The VAS sweet spot was near the DRTT in the dorsomedial region of the VIM, whereas the VAS sour spot deviated ventral and posterior from the DRTT (Fig. 2D).

### Effect of Ring Level and Amplitude on the Subjective Patient's Rating

**FIG. 3:**
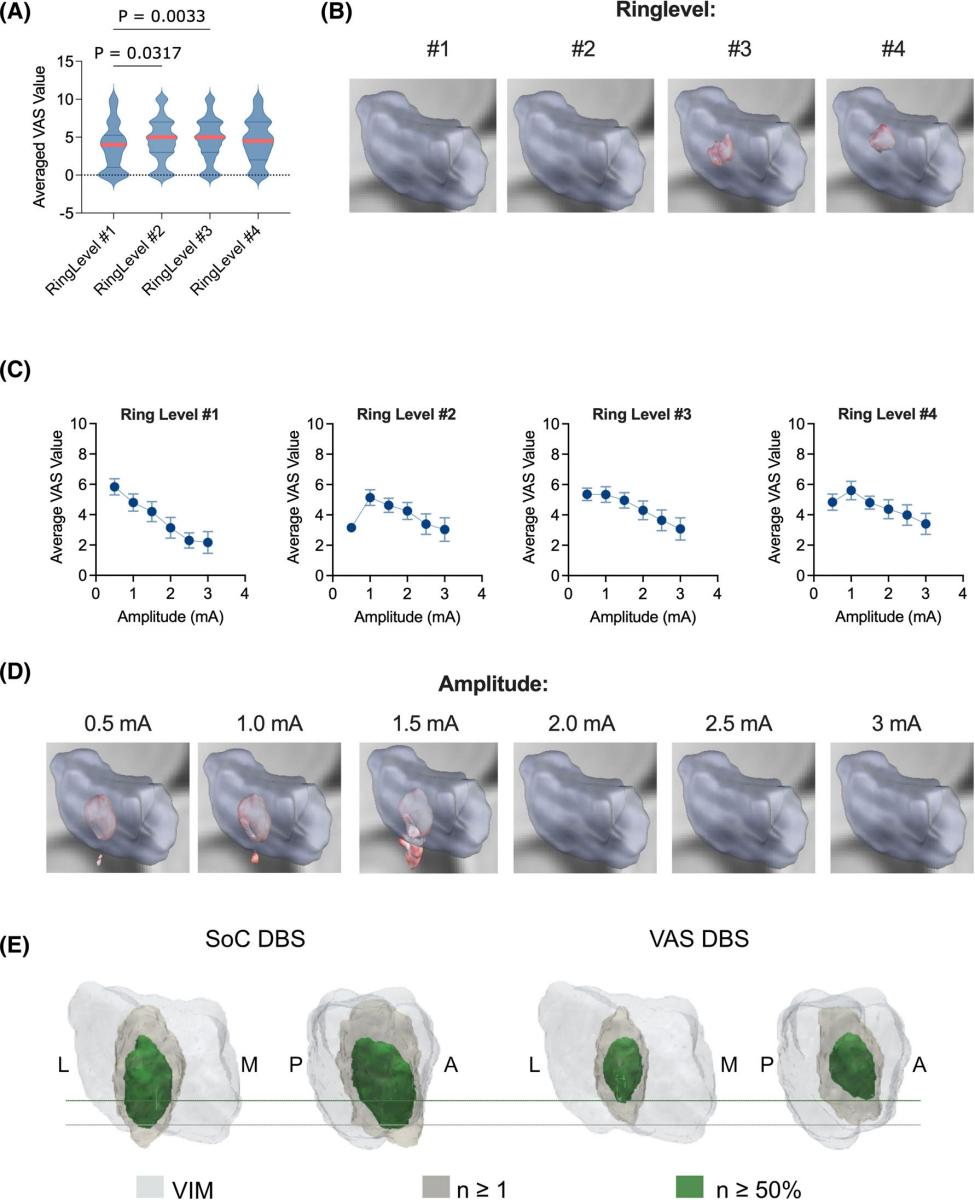
Significance of the electrode position, ring level, and amplitude on subjective ratings. (**A**) Violin plot illustrating the correlation of electrode ring level (1–4) with the average Visual Analogue Scale (VAS) rating. (**B**) Microimages illustrating the significant VAS sweet spots (*T*‐values, *P* < 0.05) at different ring levels. (**C**) Graphs illustrating the impact of stimulation amplitude on VAS scores for each ring level separately. (**D**) Microimages illustrating the significant VAS sweet spots (*T*‐values, *P* < 0.05) at different stimulation amplitudes. VAS scores across ring levels were analyzed using one‐way ANOVA (analysis of variance). (**E**) Voxel‐wise overlap (n‐maps) of volumes of tissue activated (VTA) was computed in common Montreal Neurological Institute (MNI) space for the VAS and standard‐of‐care (SoC) conditions. The ventral intermediate nucleus (VIM) nucleus is shown as anatomical reference (light gray). Voxels covered by at least one VTA (n ≥ 1) are shown in beige, and voxels covered by at least 50% of hemispheres (n > 50%) are shown in green. A, anterior; L, lateral; M, medial; P, posterior. [Color figure can be viewed at wileyonlinelibrary.com]

To further examine the relationship between subjective ratings and VTA localization, VAS scores were compared across ring levels using one‐way ANOVA. A significant main effect of ring level was observed (*F*(3,1220) = 4.06, *P* = 0.0069, *R* ^2^ = 0.0099), indicating that subjective ratings differed as a function of stimulation depth. Homogeneity of variances was confirmed by both the Brown–Forsythe test (*P* = 0.33) and Bartlett's test (*P* = 0.53). Post hoc Tukey's multiple comparisons revealed significantly lower VAS scores at the first (ventral) ring level compared to the second (mean difference = −0.70, *P* = 0.0317) and third ring levels (mean difference = −0.88, *P* = 0.0033). Consistent with these findings, ratings were significantly higher at the second and third ring levels compared to the ventral level (Fig. 3A), and statistically significant “sweet spots” were identified at the third and fourth levels (Fig. 3B). With respect to stimulation amplitude, average VAS ratings peaked at 0.5 to 1 mA and declined at higher intensities (Fig. 3C). Therefore, significant sweet spots were detected at 0.5, 1, and 1.5 mA, whereas statistical significance was no longer observed at higher amplitudes (Fig. 3D). Voxel‐wise overlap analyses (n‐maps) of the VTAs for the VAS and SoC conditions demonstrated that VAS VTAs were predominantly located in a more dorsal region of the VIM (Fig. 3E).

### Connectivity Patterns Associated with Patient‐Reported Feedback

**FIG. 4:**
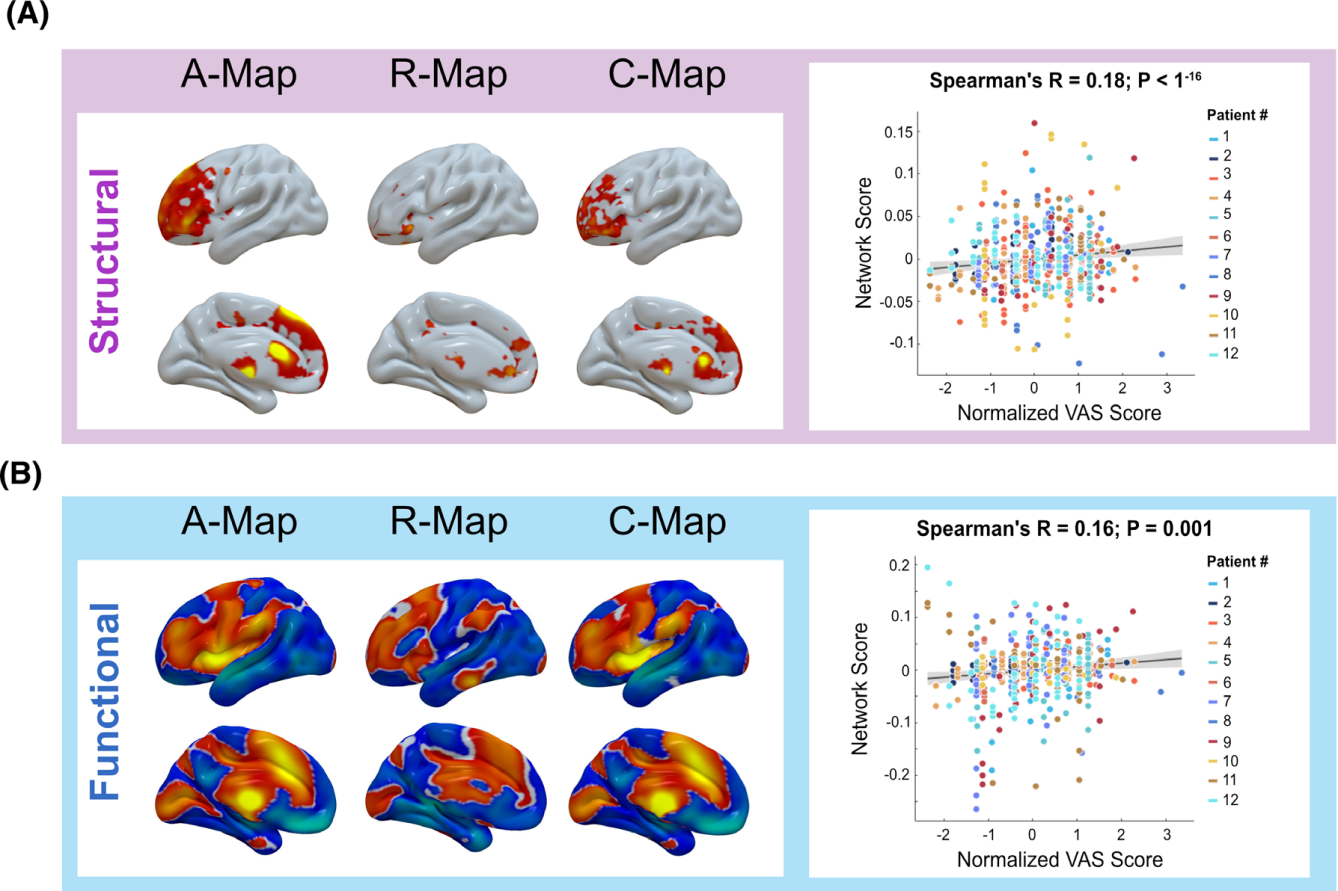
Patient subjective ratings correlate with beneficial brain network connectivity. (**A**) Structural and (**B**) functional connectivity profiles associated with subjective benefit from stimulation. Shown are weighted average maps (A‐maps, left column), correlation maps relating voxel‐wise connectivity strength to Visual Analogue Scale (VAS) ratings (R‐maps, middle column), and combined maps (C‐maps, right column) integrating both approaches. Warm colors indicate regions where stronger connectivity of the volumes of tissue activated (VTA) was associated with higher VAS scores, whereas cool colors indicate regions associated with lower scores. Predictive validity was assessed using a leave‐one‐cohort‐out cross‐validation procedure, in which network similarity scores derived from the C‐map significantly correlated with individual VAS ratings. [Color figure can be viewed at wileyonlinelibrary.com]

Stimulation sweet spots are believed to connect to various remote brain networks^17^, ^38^ that patients may perceive as either favorable or unfavorable. To explore the brain network associated with subjective patient rating,^39^ we examined the connectome profiles of our subjective sweet spots using whole‐brain structural and functional connectivity seeding from each individual VTAs. Structural connectivity demonstrated positive association between VTAs with a high VAS score in the prefrontal and frontal lobes, mainly the superior and inferior frontal gyri (Fig. 4A). As for functional connectivity, it exhibited beneficial connectivity profile that was largely similar to those of structural connectivity, with additional connectivity to the insular cortex (Fig. 4B). These models were further validated by employing a leave‐one‐cohort‐out strategy (functional: *R* = 0.18, *P* < 1^−16^; structural: *R* = 0.16, *P* = 0.001).

## Discussion

### Short‐Term Clinical Efficacy of VAS‐Based Programming

Consistent with previous findings,^23^ no significant differences were observed between VAS‐based and standard programming (Fig. 1A,B), supporting subjective patient feedback as a valid DBS programming signal. VAS‐based settings achieved similar clinical outcomes with lower amplitudes (Fig. 1C), which is relevant because higher amplitudes enlarge the VTA and increase the risk of cerebellar‐related side effects such as dysarthria, gait disturbance, and ataxia.^12^, ^40^, ^41^ Patients consistently preferred these lower‐amplitude settings (Fig. 1E), suggesting that subjective feedback can detect benefits and early side‐effect signals before overt motor changes arise. Thus, VAS‐based programming may complement SoC or emerging image‐guided or Local Field Potential (LFP)‐based approaches^42^ by supporting individualized, amplitude‐sparing programming and potentially improving tolerability, consistent with recent evidence of reliable VAS ratings in STN‐DBS.^43^

### Subjective Sweet and Sour Spots

Treatment success in ET‐DBS is largely determined by choosing the most effective target structure. Whereas the VIM has traditionally been regarded as an effective target for tremor control,^44^ more recent results suggest stimulation sites ventral to the VIM to be most effective (Fig. S3).^11^, ^17^, ^38^, ^45^, ^46^ Some studies imply that the proximity of the VTA to the DRTT was associated with greater tremor‐suppression efficiency^12^, ^13^ and that the distance to the DRTT is more critical for clinical efficacy than specific coordinates,^14^, ^15^, ^16^, ^47^ suggesting that different target regions may represent a common anatomical fiber tract—the DRTT.^18^, ^48^ In accord with this, the PSA, including the cZi, Forel field H, and the prelemniscal radiation, was proposed as an effective and alternative stimulation target for ET. In fact, several studies proposed that PSA‐DBS might have better efficacy in controlling tremor symptoms and cause fewer stimulation‐related side effects.^12^, ^49^, ^50^, ^51^, ^52^, ^53^ Other studies have also postulated the existence of stimulation sweet spots more anteriorly in the region of the ventralis oralis posterior (VOp) nucleus or along the VIM/VOp border.^38^, ^54^, ^55^ By pairing VAS scores with VTAs, we identified regions with the highest and lowest subjective ratings—termed the “subjective sweet” and “sour” spots. The sweet spot localized dorsally within the VIM, whereas the sour spot lay posteroventrally, below the VIM (Fig. 2B). This contrasts with reports placing the tremor sweet spot more ventrally, possibly due to side effects or negative subjective sensations not evident in clinical exams. The VAS sweet spot was near the DRTT, whereas the sour spot was farther from its ventral entry into the VIM, consistent with effective tremor control involving DRTT engagement (Fig. 2D). Overall, our findings emphasize incorporating patient feedback when selecting ventral contacts in VIM‐DBS and support the concept of outcome‐specific sweet spots, as recently described in PD.^56^ Future prospective studies should compare these distinct sweet spots in terms of clinical efficacy and patient satisfaction in chronic ET‐DBS. Ring‐level analyses indicated a dorsally shifted preference, with higher VAS ratings at the second to third rings and sweet spots at the third to fourth rings near the dorsal VIM/DRTT, and lower amplitudes yielding more focal VTAs and significant sweet spots (Fig. 3A,B,D). Although SoC programming here followed conventional symptom–side‐effect mapping and imaging, emerging image‐guided, connectivity‐informed, and LFP‐based methods—and ongoing work linking VAS outcomes to LFP biomarkers—may soon support more objective, physiologically informed individualized programming.

### Connectivity of Subjective Sweet Spots in ET

Recent diffusion tensor imaging (DTI)‐based connectivity studies on thalamic DBS suggest that the cerebello‐thalamo‐cortical network plays a key role in tremor modulation.^57^ Strong connectivity between active DBS contacts and network nodes is assumed to be linked to therapeutic effects. Some studies focused on specific network nodes,^58^ whereas others analyzed whole‐brain connectivity using patient‐specific or normative connectome data.^17^, ^45^ For instance, Akram et al. used probabilistic tractography to show high structural connectivity between the VIM and M1, Supplementary Motor Area (SMA), S1, and contralateral dentate nucleus.^45^ Grimm et al. examined 20 ET patients who had undergone bilateral DBS using patient‐specific probabilistic diffusion tensor imaging, identifying that the connectivity between M1 and the somatosensory cortex was most closely related to complete and incomplete tremor suppression, with the anterior lobe of the cerebellum and SMA also involved.^59^ Similarly, Al‐Fatly et al. identified the patterns of effective VIM‐DBS connectivity by normative brain connectomes and found that there was positive connectivity in multiple regions, mainly in the paracentral gyrus, visual cortex, and superior and inferior cerebellar lobules.^11^, ^17^ In accord with previous studies,^57^, ^60^ the subjective VAS sweet spot identified in our study was located close to the DRTT. VTAs associated with higher VAS ratings additionally exhibited connectivity to frontal, prefrontal, and insular regions (Fig. 4). These regions are implicated in cognitive, affective, and interoceptive processing^61^, ^62^ and may therefore reflect higher‐order appraisal of stimulation effects rather than direct modulation of tremor severity. For instance, the insula—especially the anterior insula—integrates interoceptive, affective, and salience‐related information underlying conscious bodily awareness and subjective stimulus evaluation,^63^, ^64^, ^65^ and thus its connectivity could reflect the perceptual–evaluative processes captured by VAS ratings rather than direct tremor suppression, including possible expectation‐driven (placebo‐like) influences.^66^ Particularly, patients preferred lower amplitudes and smaller VTAs, suggesting that subjective ratings may be influenced not only by engagement of beneficial networks but also by the avoidance of stimulation of nonmotor or side effect–related circuits. However, formally assessing the absence of connectivity or disentangling these effects is methodologically challenging and beyond the scope of the present study. Therefore, the connectivity findings should be considered hypothesis generating and secondary to the primary VAS–VTA results, serving mainly to inform future work aimed at integrating subjective feedback with network‐level models of DBS effects.

## Limitations

This study has several limitations. First, all data were collected in an acute setting, capturing only short‐term effects; therefore, the long‐term clinical value of VAS‐based programming remains unknown. Although tremor typically responds to DBS within seconds, brief washout intervals may still leave minor residual effects. Future studies with repeated assessments or extended follow‐up will be needed to evaluate the reproducibility and durability of VAS‐guided programming. Second, VAS ratings are subjective and may be influenced by cognitive or affective factors. Our protocol—using blinded stimulation parameters, randomized contact–amplitude combinations, and washout intervals—was designed to minimize habituation and expectancy effects, but some residual influence is unavoidable. Combining subjective ratings with objective clinical or biomarker measures may provide a more comprehensive evaluation. Third, the sample size was small and limited to a single center, which constrains generalizability and may reduce power in spatial analyses such as sweet‐spot mapping. Larger, multicenter cohorts will be essential for validation and subgroup analyses across DBS targets or patient populations. Finally, because the sweet spots in the thalamic and subthalamic regions are small—affecting the validity of the connectome analyses and resulting in low *R* values—these analyses should be regarded as exploratory.

## Conclusion

Our findings indicate that VAS‐guided patient feedback is a valuable tool for optimizing and personalizing DBS programming in ET, linking perceived benefit to sensorimotor network connectivity and warranting further evaluation in larger, diverse cohorts. Although the current study addresses only acute effects, repeated VAS assessments could be leveraged in the future as part of “subjective closed‐loop” or semiautomated DBS algorithms—for example, through smartphone‐based patient polling—to support ongoing parameter optimization in real‐world settings.

## Author Roles

J.D., S.P., C.P., E.K., and T.K. designed the experiments; S.P., A.K., J.O., J.H.M., J.S., and J.D. performed the experiments. S.P., J.D., M.S., and T.K. analyzed the data. J.D., S.P., and T.K. wrote the manuscript. E.K., J.D., M.S., C.P., and T.K. edited the final version of the manuscript.

## Financial Disclosures and Conflicts of Interest

Author disclosures are available in the Supporting Information.

## Full financial disclosures of all authors for the past 12 months

J.D.: none. S.P.: none. A.K.: none. M.S. was supported by a Feodor‐Lynen Return‐Fellowship. C.P. was funded by Deutsche Forschungsgemeinschaft (DFG, German Research Foundation) under Germany's Excellence Strategy within the framework of the Munich Cluster for Systems Neurology (EXC 2145 SyNergy ID 390857198), the Thiemann Stiftung and Else‐Kröner‐Fresenius Stiftung. J.H.M.: none. J.O.: none. J.S.: none. E.K. was funded by the Deutsche Forschungsgemeinschaft (DFG, German Research Foundation) and the Medical & Clinician Scientist Program (MCSP). E.K. received speaker honoraria and financial compensation for travel expenses from Medtronic, UCB, Livanova, Desitin, Precisis, UNEEG, and Eisai and has participated in clinical trials for Medtronic, UCB, Ergomed, and Precisis, all unrelated to the submitted work. T.K. was funded by Parkinson Fonds Deutschland, Stichting ParkinsonFonds, Fritz Thyssen Stiftung, FRAXA, VDI, and Medical & Clinician Scientist Program (MCSP); received industry funding from Abbott Medical Inc., Medtronic, and AbbVie; served on advisory boards Mitsubishi; and received speaker honoraria and travel support for scientific presentations from Abbott Medical Inc. and AbbVie.

## Supporting information


**TABLE S1.** Spreadsheet for testing and documenting VAS scores in response to different combinations of contact and amplitude during VAS monopolar review.
**TABLE S2**. Patient demographics and DBS programming parameters. The table lists age range, gender, disease onset year, and DBS duration for each patient. Device type, stimulation frequency, pulse width, amplitude, active contacts, and ring level are provided for both pre‐ and post‐VAS assessments for left and right electrodes. C (+) indicates the cathode contact, and numbers following represent anodes or segmented contacts. Dash (“–”) indicates data not available or stimulation turned off.
**FIG. S1**: Images illustrating the VAS sweet (red) and sour (blue) spots before and after determination of statistical significance at an α‐level of 0.05.
**FIG. S2**. Two‐dimensional reconstruction of all electrode contacts (#1–8) showing their locations in normalized space. Electrodes were surgically targeted to the VIM following standard stereotactic protocol. Visual inspection confirms accurate placement, with a slight posterior bias. Variability is slightly greater on the right hemisphere, consistent with the second implanted side.
**FIG. S3**. Schematic comparison of previously defined sweet spots with the sweet and sour spots identified in our VAS‐based analysis.
**FIG. S4**. Highspatial overlap between VTAs from incremental current steps. (a, b) Example pairwise VTA containment across incremental current steps. (c) Table showing each current step and its corresponding containment value, defined as the proportion of the lower‐amplitude VTA contained within the subsequent higher‐amplitude VTA. For example, a containment value of 0.77 for 0.5 → 1.0 mA indicates that 77% of the 0.5 mA VTA is contained within the 1.0 mA VTA.

## Data Availability

The data that support the findings of this study are available from the corresponding author upon reasonable request.
