# Supplementary material for: Patient‐Reported Feedback Suggests an Alternative Sweet Spot for Deep Brain Stimulation Programming in Essential Tremor
Source: Mov Disord. 2026 Mar 6;41(5):1209–20. doi: 10.1002/mds.70228 (PMC13206464; doi:10.1002/mds.70228)
Supplement: Supplementary file 1 — TABLE S1. Spreadsheet for testing and documenting VAS scores in response to different combinations of contact and amplitude during VAS monopolar review. TABLE S2. Patient demographics and DBS programming parameters. The table lists age range, gender, disease onset year, and DBS duration for each patient. Device type, stimulation frequency, pulse width, amplitude, active contacts, and ring level are provided for both pre‐ and post‐VAS assessments for left and right electrodes. C (+) indicates the cathode contact, and numbers following represent anodes or segmented contacts. Dash (“–”) indicates data not available or stimulation turned off. FIG. S1: Images illustrating the VAS sweet (red) and sour (blue) spots before and after determination of statistical significance at an α‐level of 0.05. FIG. S2. Two‐dimensional reconstruction of all electrode contacts (#1–8) showing their locations in normalized space. Electrodes were surgically targeted to the VIM following standard stereotactic protocol. Visual inspection confirms accurate placement, with a slight posterior bias. Variability is slightly greater on the right hemisphere, consistent with the second implanted side. FIG. S3. Schematic comparison of previously defined sweet spots with the sweet and sour spots identified in our VAS‐based analysis. FIG. S4. Highspatial overlap between VTAs from incremental current steps. (a, b) Example pairwise VTA containment across incremental current steps. (c) Table showing each current step and its corresponding containment value, defined as the proportion of the lower‐amplitude VTA contained within the subsequent higher‐amplitude VTA. For example, a containment value of 0.77 for 0.5 → 1.0 mA indicates that 77% of the 0.5 mA VTA is contained within the 1.0 mA VTA. [file MDS-41-1209-s001.docx]

**Supplemental Information**

**Patient Reported Feedback Suggests an Alternative Sweet Spot for DBS Programming in Essential Tremor**

Sophia Peschke,^1,#^ Jing Dong,^1,#^ Angelina Kirschner,^1^ Johannes Off,^1^ Juhi Shaik,^1^ Dr. Carla Palleis, MD,^1^ Dr. Jan Hinnerk Mehrkens, MD,^3^ Dr. Elisabeth Kaufmann, MD,^1^ & Dr. Maximilian Scherer, PhD^1,#^ & Dr. Thomas Koeglsperger, MD,^1,2,#^

**Supplememtal Methods:**

**Study Visit and VAS Rating** Chronic stimulation parameters were recorded, and tremor severity was assessed using the Fahn-Tolosa-Marín Tremor Rating Scale (FTMTRS) prior to deactivation of bilateral stimulation. Following a 60-second washout period, FTMTRS scores were reassessed in the STIM-OFF condition. Subsequently, patients underwent VAS-based reprogramming of their stimulator, with each hemisphere tested independently while the contralateral side remained switched off. For each hemisphere, different stimulation amplitudes (ranging from 0.5 to 3.0 mA, including a 0mA – DBS OFF – control) were applied individually to each contact. The sequence of contact-amplitude combinations was randomized using a predefined list to minimize habituation effects (**Supp. Table 1, Supp. Fig. 4**). Following each adjustment, patients were asked to rate the overall quality of the DBS effect on a scale from 0 to 10, where 0 indicated "very bad" and 10 indicated "very good," without further elaboration. Intermediate scores were left to the patients' interpretation, based purely on their subjective perception of the stimulation effect. Patients were instructed to provide a rating within 10-15 seconds, a time window deemed sufficient for symptom modulation to occur.^1^ The corresponding VAS score was recorded immediately after each setting. Throughout the procedure, patients were blinded to the specific stimulation settings. If intolerable side effects emerged, the corresponding contact was excluded from further testing at higher amplitudes. After each stimulation setting, a 10-second washout period was maintained before the next parameter combination was applied.^1–3^ Once one hemisphere was completed, the same procedure was repeated for the contralateral side. The overall VAS-based adjustment process required approximately 60 minutes per electrode per patient. Following completion of testing, the best-rated stimulation setting (combination of contact and amplitude with the highest VAS rating, PW = 60 μs; f = 130 Hz) for each hemisphere was selected. Stimulation was then reinitiated bilaterally using the chosen settings, and after a three-minute habituation period, final FTMTRS assessments were conducted. All participants were returned to their original settings at the conclusion of the study visit.

**Supplemental Table 1**

| **LEFT ELECTRODE** | | |  | **RIGHT ELECTRODE** | | |
| --- | --- | --- | --- | --- | --- | --- |
| **Contact** | **Amplitude** | **VAS** |  | **Contact** | **Amplitude** | **VAS** |
| 4 | 2,5 |  |  | 9 | 2 |  |
| 4 | 1,5 |  |  | 9 | 2,5 |  |
| 4 | 3 |  |  | 12 | 2 |  |
| 1 | 1,5 |  |  | 9 | 1,5 |  |
| 3C | 3 |  |  | 12 | 1,5 |  |
| 1 | 0,5 |  |  | 9 | 0,5 |  |
| 1 | 1 |  |  | 10A | 2,5 |  |
| 2A | 1 |  |  | 10A | 2 |  |
| 2C | 3 |  |  | 11A | 3 |  |
| 2A | 2,5 |  |  | 10B | 1,5 |  |
| 3C | 2,5 |  |  | 10A | 1,5 |  |
| 2A | 0,5 |  |  | 10A | 0,5 |  |
| 1 | 2,5 |  |  | 12 | 1 |  |
| 3A | 1,5 |  |  | 10B | 1 |  |
| 2A | 1,5 |  |  | 11A | 2 |  |
| 3C | 2 |  |  | 10C | 1 |  |
| 2B | 2,5 |  |  | 9 | 1 |  |
| 2C | 2 |  |  | 11B | 1 |  |
| 1 | 2 |  |  | 10C | 2,5 |  |
| 2A | 2 |  |  | 11B | 2 |  |
| 2C | 1,5 |  |  | 10A | 3 |  |
| 2B | 0,5 |  |  | 10B | 0,5 |  |
| 3A | 2 |  |  | 11C | 3 |  |
| 2B | 1 |  |  | 10C | 1,5 |  |
| 3A | 1 |  |  | 11B | 3 |  |
| 2C | 0,5 |  |  | 10C | 0,5 |  |
| 1 | 3 |  |  | 9 | 3 |  |
| OFF | 0 |  |  | OFF | 0 |  |
| 2C | 2,5 |  |  | 11A | 2,5 |  |
| 3B | 1 |  |  | 10C | 3 |  |
| 2A | 3 |  |  | 11B | 1,5 |  |
| 3B | 1,5 |  |  | 10B | 2 |  |
| 2B | 2 |  |  | 11B | 2,5 |  |
| 3B | 2,5 |  |  | 12 | 2,5 |  |
| 3A | 0,5 |  |  | 11A | 0,5 |  |
| 2B | 3 |  |  | 10B | 2,5 |  |
| 4B | 0,5 |  |  | 11B | 0,5 |  |
| 3B | 3 |  |  | 10A | 1 |  |
| 3A | 2,5 |  |  | 11A | 1 |  |
| 3C | 1,5 |  |  | 10B | 3 |  |
| 2C | 1 |  |  | 11C | 2 |  |
| 3A | 3 |  |  | 11A | 1,5 |  |
| 3C | 0,5 |  |  | 11C | 0,5 |  |
| 2B | 1,5 |  |  | 11C | 2,5 |  |
| 3C | 1 |  |  | 11C | 1,5 |  |
| 3B | 2 |  |  | 10C | 2 |  |
| 4 | 1 |  |  | 12 | 3 |  |
| 4 | 2 |  |  | 11C | 1 |  |
| 4 | 0,5 |  |  | 12 | 0,5 |  |

**Supplemental Table 1:** Spreadsheet for testing and documenting VAS scores in response to different combinations of contact and amplitude during VAS monopolar review.

**Supplemental Table 2**

| Pat. no. | Age  range  (yrs) | Gender | Disease  onset  (yr) | DBS  duration  (yrs) | Device | Frequency  Pre-VAS  (Hz) | Frequency  Post-VAS  (Hz) | Pulse  width  Pre-VAS  (μs) | Pulse  width  Post-VAS  (μs) | Amplitude  Pre-VAS  (mA) | Amplitude  Post-VAS  (mA) | Contact  Pre-VAS | Contact  Post-VAS | Ringlevel  Pre-VAS | Ringlevel  Post-VAS |
| --- | --- | --- | --- | --- | --- | --- | --- | --- | --- | --- | --- | --- | --- | --- | --- |
| **LEFT ELECTRODE** | | | | | | | | | | | | | | | |
| 1 | 60-65 | m | 2000 | 5 | Boston | 185 | 130 | 60 | 60 | 3.2 | 2.5 | C (+); 4/7 (-) | C (+); 4 (-) | 2/3 | 2 |
| 2 | 65-70 | w | 1999 | 11 | Medtronic | 130 | - | 60 | 60 | 5.5 | - | 10 (+); 9 (-) | - | 2 | - |
| 3 | 70-75 | m | 2008 | 6 | Boston | 185 | OFF | 30 | 60 | 3.3 | OFF | C (+); 6 (-) | OFF | 3 | OFF |
| 4 | 75-80 | m | 1965 | 2 | Abbott | 180 | 130 | 30 | 60 | 4.0 | 1.0 | C (+); 2 A/B (-) | C (+); 1 (-) | 2 | 1 |
| 5 | 65-70 | w | 1995 | 11 | Medtronic | 180 | 130 | 60 | 60 | 7.0 | 1.5 | C (+); 0 (-) | C (+); 0 (-) | 1 | 1 |
| 6 | 60-65 | w | 2018 | 2 | Boston | 185 | 130 | 30 | 60 | 4.9 | 2.5 | C (+); 1 (-) | C (+); 5 (-) | 1 | 3 |
| 7 | 60-65 | m | 2003 | 1 | Boston | 159 | 130 | 40 | 60 | 6.5 | 1.0 | 5 (+); 4 (-) | C (+); 5 (-) | 2 | 3 |
| 8 | 75-80 | m | 2008 | 5 | Boston | 149 | 130 | 30 | 60 | 5.0 | 2.0 | C (+); 4/7 (-) | C (+); 4 (-) | 2/3 | 2 |
| 9 | 80-85 | m | 1970 | 20 | Medtronic | 130 | 130 | 90 | 60 | 4.0 | 3.0 | C (+); 0 (-) | C (+); 0 (-) | 1 | 1 |
| 10 | 80-85 | m | 1995 | 6 | Boston | 198 | 130 | 30 | 60 | 4.4 | 2.0 | C (+); 1/2 (-) | C (+); 2 (-) | 1/2 | 2 |
| 11 | 75-80 | m | 2005 | 10 | Boston | 119 | 130 | 60 | 60 | 7.0 | 3.0 | 1 (+); 2/3 (-) | C (+); 8 (-) | 2 | 4 |
| 12 | 70-75 | w | 1985 | 6 | Boston | 185 | 130 | 60 | 60 | 0.1 | 1.0 | 8 (+); 1 (-) | C (+); 7 (-) | 1 | 3 |
| 13 | 65-70 | w | 1968 | 2 | Boston | 130 | 130 | 60 | 60 | 2.5 | 1.0 | C (+); 2/3/4 (-) | C (+); 8 (-) | 2 | 4 |
| 14 | 70-75 | m | 2003 | 15 | Medtronic | 130 | 130 | 60 | 60 | 2.9 | 3.0 | C (+); 1 (-) | C (+); 1 (-) | 2 | 2 |
| 15 | 80-85 | w | 1989 | 15 | Medtronic | 180 | 130 | 60 | 60 | 2.2 | 1.0 | C (+); 2 (-) | C (+); 3 (-) | 3 | 4 |
| 16 | 55-60 | m | 1970 | 5 | Boston | 130 | 130 | 60 | 60 | 3.8 | 1.5 | C (+); 2/3/4 (-) | C (+); 3 (-) | 2 | 2 |
| **RIGHT ELECTRODE** | | | | | | | | | | | | | | | |
| 1 | 60-65 | m | 2000 | 5 | Boston | 185 | 130 | 50 | 60 | 10.0 | 3.0 | 6 (+); 3 (-) | C (+); 3 (-) | 2 | 2 |
| 2 | 65-70 | w | 1999 | 11 | Medtronic | 130 | - | 60 | - | 7.5 | - | 1 (+); 2 (-) | - | 3 | - |
| 3 | 70-75 | m | 2008 | 6 | Boston | 185 | 130 | 30 | 60 | 2.5 | 1.0 | C (+); 8 (-) | C (+); 8 (-) | 4 | 4 |
| 4 | 75-80 | m | 1965 | 2 | Abbott | 180 | 130 | 30 | 60 | 4.5 | 2.0 | C (+); 11 A/B/C (-) | C (+); 11 C (-) | 3 | 3 |
| 5 | 65-70 | w | 1995 | 11 | Medtronic | 180 | 130 | 60 | 60 | 6.6 | 3.0 | C (+); 9 (-) | C (+); 9 (-) | 2 | 2 |
| 6 | 60-65 | w | 2018 | 2 | Boston | 185 | 130 | 30 | 60 | 7.5 | 1.0 | 5 (+); 2 (-) | C (+); 5 (-) | 2 | 3 |
| 7 | 60-65 | m | 2003 | 1 | Boston | 159 | 130 | 40 | 60 | 12.0 | 0.5 | 8/1 (+); 6 (-) | C (+); 4 (-) | 2 | 2 |
| 8 | 75-80 | m | 2008 | 5 | Boston | 149 | 130 | 30 | 60 | 2.6 | 1.0 | C (+); 8 (-) | C (+); 2 (-) | 4 | 2 |
| 9 | 80-85 | m | 1970 | 20 | Medtronic |  |  |  |  |  |  |  |  |  |  |
| 10 | 80-85 | m | 1995 | 6 | Boston | 198 | 130 | 40 | 60 | 5.2 | 3.0 | C (+); 1 (-) | C (+); 4 (-) | 1 | 2 |
| 11 | 75-80 | m | 2005 | 10 | Boston | 119 | 130 | 60 | 60 | 8.5 | 3.0 | 1 (+); 2 (-) | C (+); 8 (-) | 2 | 4 |
| 12 | 70-75 | w | 1985 | 6 | Boston | 185 | 130 | 30 | 60 | 5.0 | 2.0 | C (+); 2/5 (-) | C (+); 8 (-) | 2/3 | 4 |
| 13 | 65-70 | w | 1968 | 2 | Boston | 130 | 130 | 60 | 60 | 1.7 | 0.5 | C (+); 2/3/4 (-) | C (+); 1 (-) | 2 | 1 |
| 14 | 70-75 | m | 2003 | 15 | Medtronic | 130 | 130 | 60 | 60 | 3.3 | 3.0 | C (+); 9 (-) | C (+); 9 (-) | 2 | 2 |
| 15 | 80-85 | w | 1989 | 15 | Medtronic | 180 | 130 | 60 | 60 | 0.8 | 0.5 | C (+); 9 (-) | C (+); 10 (-) | 2 | 3 |
| 16 | 55-60 | m | 1970 | 5 | Boston | 130 | 130 | 60 | 60 | 3.7 | 3.0 | C (+); 2/3/4 (-) | C (+); 4 (-) | 2 | 2 |

**Supplemental Table 2:** Patient demographics and DBS programming parameters. The table lists age range, gender, disease onset year, and DBS duration for each patient. Device type, stimulation frequency, pulse width, amplitude, active contacts, and ring level are provided for both pre- and post-VAS assessments for left and right electrodes. C (+) indicates the cathode contact, and numbers following represent anodes or segmented contacts. Dash (“–”) indicates data not available or stimulation turned off.

**Supplemental Figure 1**

**^
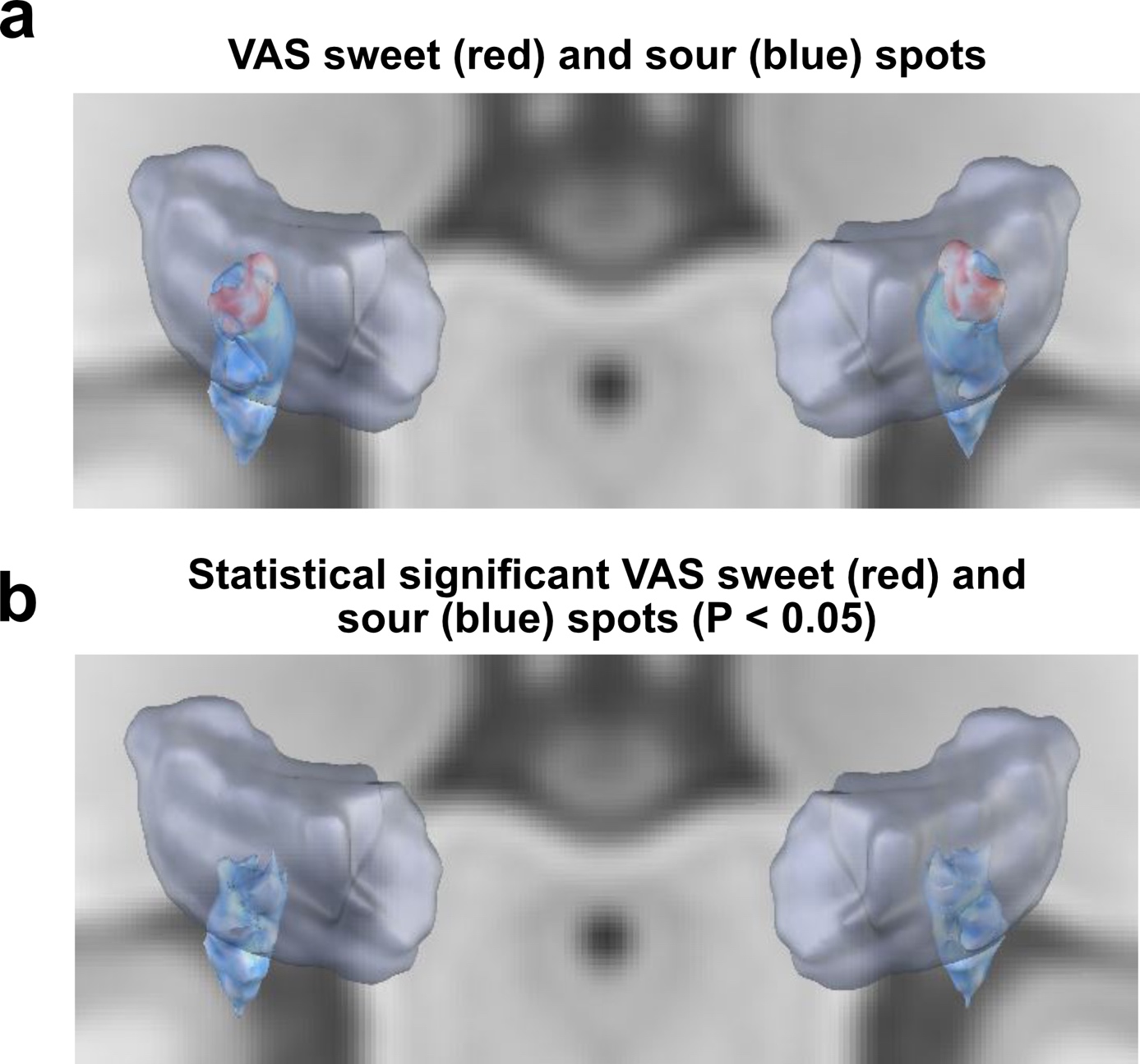
^**

**Supplemental Figure 1**: Images illustrating the VAS sweet (red) and sour (blue) spots before and after determination of statistical significance at an α-level of 0.05.

**Supplemental Figure 2**

**
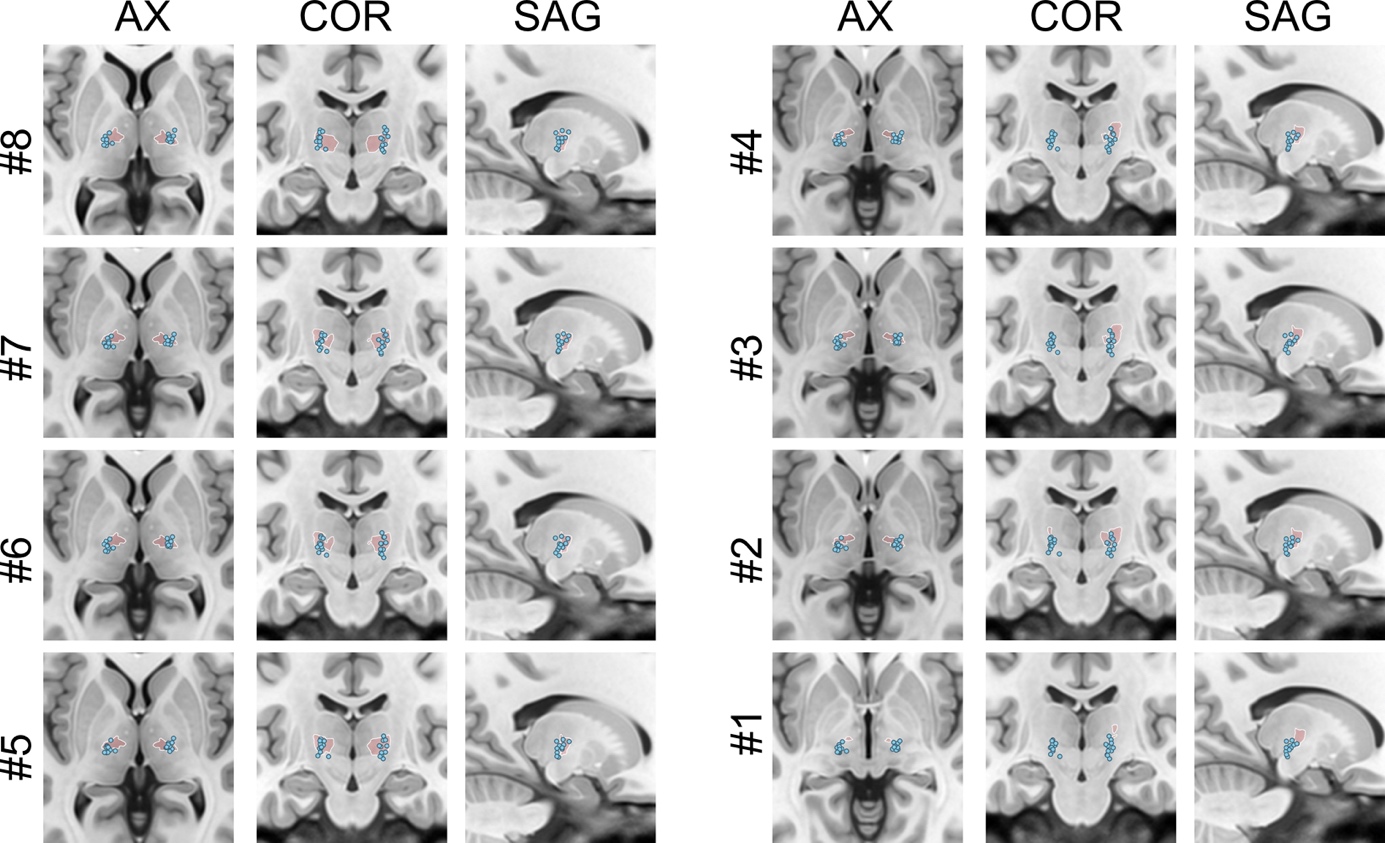
**

**Supplemental Figure 2:** Two-dimensional reconstruction of all electrode contacts (#1–8) showing their locations in normalized space. Electrodes were surgically targeted to the VIM following standard stereotactic protocol. Visual inspection confirms accurate placement, with a slight posterior bias. Variability is slightly greater on the right hemisphere, consistent with the second implanted side.

**Supplemental Figure 3**

**
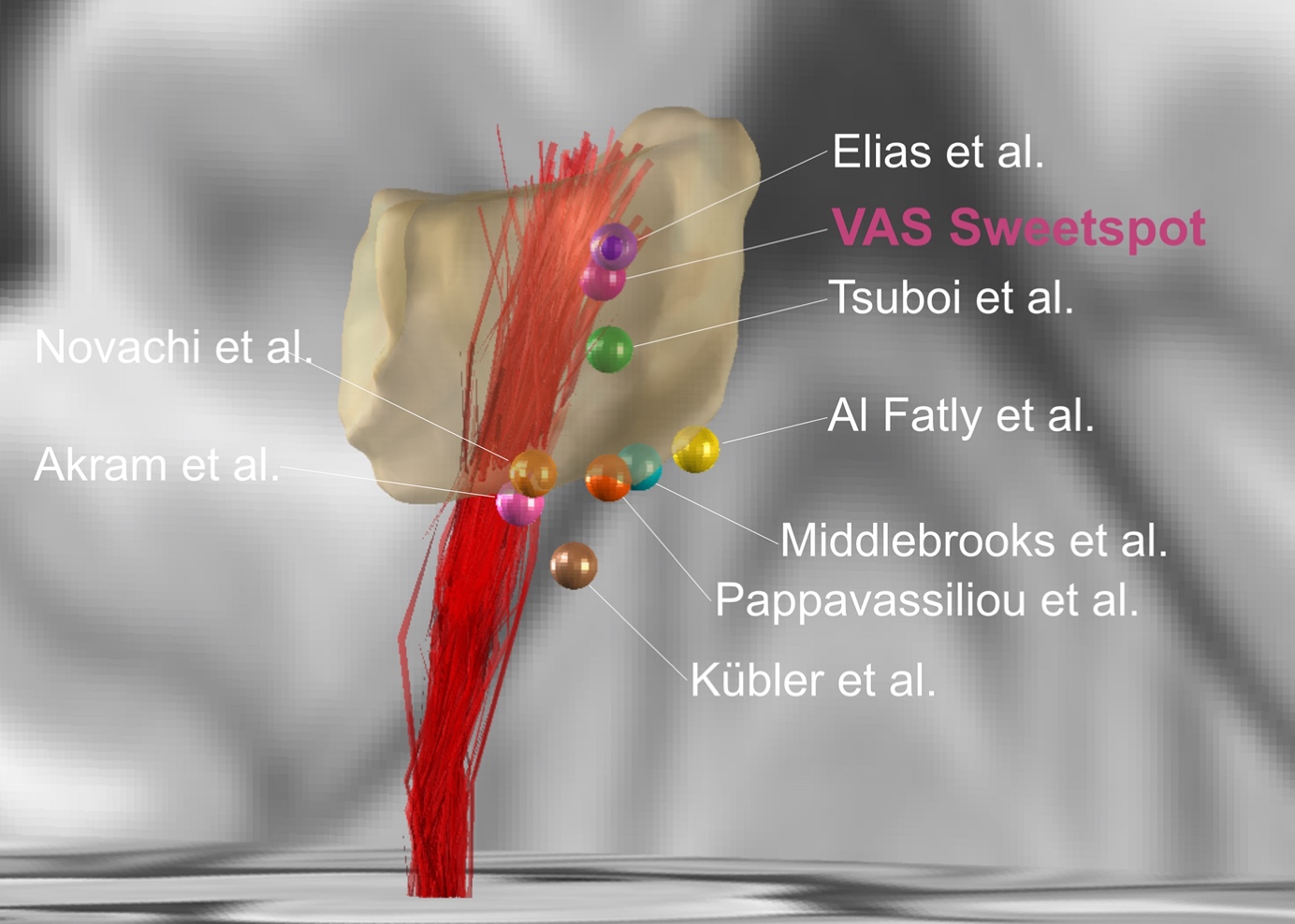
**

**Supplemental Figure 3:** Schematic comparison of previously defined sweet spots with the sweet and sour spots identified in our VAS-based analysis.

**Supplemental Figure 4**

**
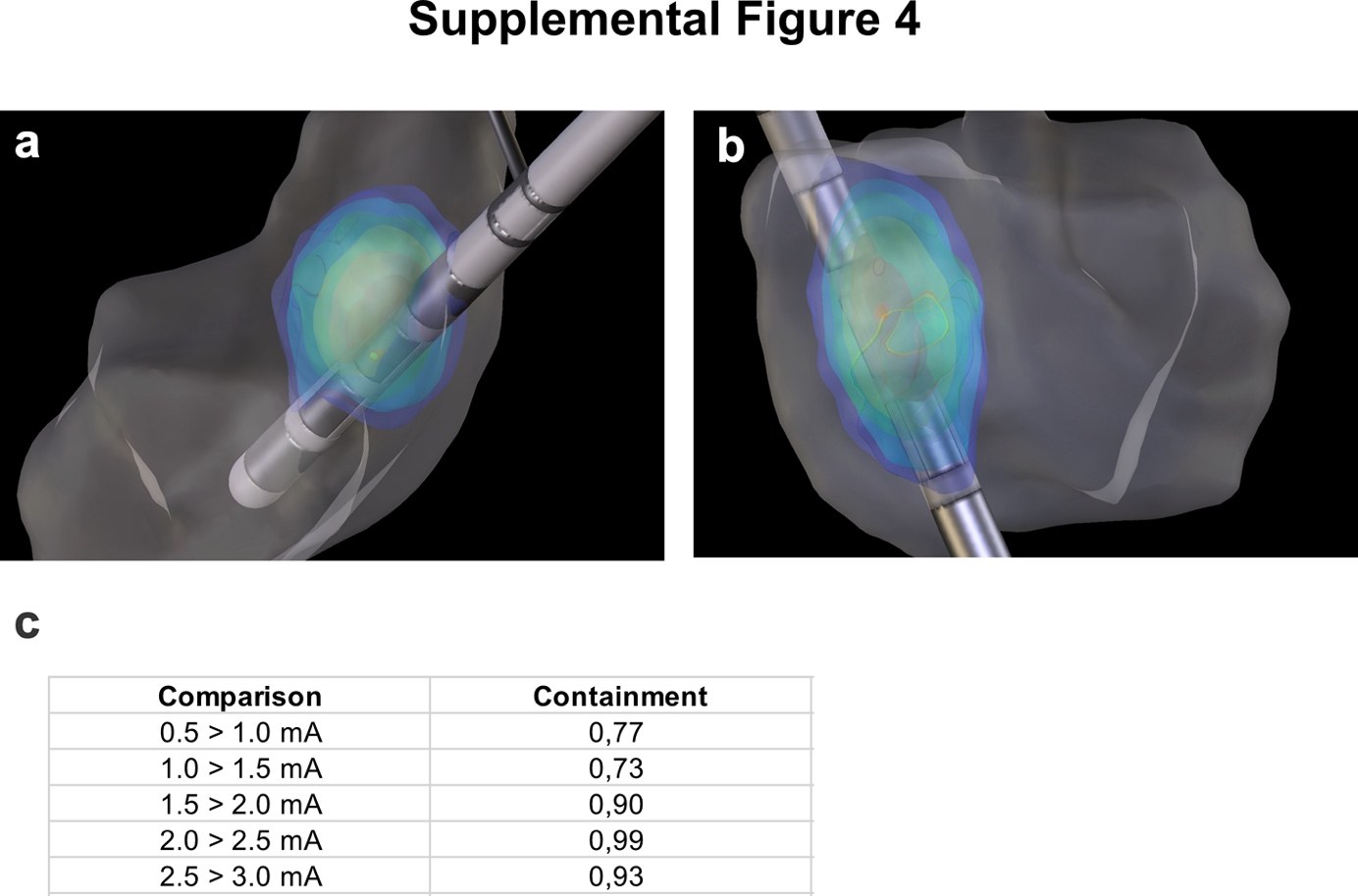
**

**Supplemental Figure 4 High spatial overlap between VTAs from incremental current steps.** (**a, b**) Example pairwise VTA containment across incremental current steps.
(**c**) Table showing each current step and its corresponding containment value, defined as the proportion of the lower-amplitude VTA contained within the subsequent higher-amplitude VTA. For example, a containment value of 0.77 for 0.5 → 1.0 mA indicates that 77% of the 0.5 mA VTA is contained within the 1.0 mA VTA.

**References:**

1. McIntyre, C. C. & Anderson, R. W. Deep brain stimulation mechanisms: the control of network activity via neurochemistry modulation. *Journal of Neurochemistry* **139**, 338–345 (2016).

2. Palleis, C., Gehmeyr, M., Mehrkens, J. H., Bötzel, K. & Koeglsperger, T. Establishment of a Visual Analog Scale for DBS Programming (VISUAL-STIM Trial). *Front Neurol* **11**, 561323 (2020).

3. Dong, J. *et al.* Subjective Patient Rating as a Novel Feedback Signal for DBS Programming in Parkinson’s Disease. *Brain Stimul.* (2025) doi:10.1016/j.brs.2025.03.008.
